# Medication belief as correlate of medication adherence among patients with diabetes in Edo State, Nigeria

**DOI:** 10.1002/nop2.199

**Published:** 2018-09-14

**Authors:** Olaolorunpo Olorunfemi, Foluso Ojewole

**Affiliations:** ^1^ School of Nursing University of Benin Teaching Hospital Benin Edo State Nigeria; ^2^ Department of Nursing Babcock University Ilisha Remo Ogun State Nigeria

**Keywords:** medication adherence patient with diabetes, medication belief, nurses, nursing, re‐admission rate

## Abstract

**Aim:**

The study aim was to determine the level of medication adherence, patient medication belief and to determine the correlation between medication belief and medication adherence among patients with diabetes. This is to find out whether medication belief of patient could enhance medication adherence which in turn would promote effective management of diabetic mellitus in Nigeria.

**Background:**

The World Health Organization (WHO, 2015) estimated that over 366 million people are affected. The government and other non‐governmental organizations such as Diabetic Association of Nigeria (DAN) put several programmes in place to reduce the incidence of the disease, but minimal progress has been recorded, and the factors responsible for that is a big dilemma in the heart of researchers in the field. Empirical findings showed that, there is increased rate of re‐admission among DM patients and this is associated with poor medication adherence. There is a need to examine the factors responsible for poor medication adherence among patients with diabetes. In the review of the literature, medication belief is one of the major implicated factors, but there is no substantial evidence‐based research to validate this presumption in Nigeria. Therefore, this study is to find out the relationship between medication belief and medication adherence among patients with diabetes.

**Design:**

A correlational research design was adopted, to enable the researcher in determining the association between the medication belief and medication adherence among patients with diabetes mellitus.

**Methods:**

A total enumeration was adopted for the study, where all the registered adult patients were invited to join the study voluntarily and informed verbal consent was obtained, after explaining the importance of the study. A total of 180 patients with diabetes participated in the study. The Beliefs about Medicines Questionnaires (BMQ) and Morisky Medication Adherence Scale were used. This is a standardized scale and well validated with a reliability coefficient of 0.86, using split‐half model. A simple frequency distribution table and Spearman's rho correlational statistic were used to test the hypothesis at 0.05 level of significance.

**Results:**

The data analysis shows that the clients had a negative perception about medication and believed that medication had the tendency of causing harm or poison to their system. The correlation between medication belief and medication adherence showed *p* = 0.005 which means that there is a statistical relationship between the two variables.

## 1 INTRODUCTION

Diabetes mellitus (DM) is a chronic medical illness that poses a lot of health burdens on people. In 2015, there was estimation of about 1.702,900 million diabetic cases in Nigeria, with different types of complications such as peripheral vascular disease, retinopathy, kidney disease, neuropathy, diabetic ulcers and encephalopathy (International Diabetes Federation [IDF], 2015). Diabetes mellitus and its associated complications remain a major global concern. An estimation of 366 million people worldwide is suffering from diabetes or diabetes‐related diseases (International Diabetes Federation [IDF], 2015). This increasing incidence of diabetes mellitus imposes a large economic burden on individuals, healthcare team and other personnel involved in the healthcare system (IDF, 2013). For some years now, the trend of diabetic diseases has been on the high side, over 1.702,900 million diabetes cases in Nigeria as of 2015 (International Diabetes Federation, 2016).

Rumsfeld et al. (2009) carried out a research on 11,532 clients with diabetes mellitus in tertiary care hospitals. The study focused on the proportion of days when patients were involved in taking care of themselves by taking their prescribed oral hypoglycaemic medications and other medication adherences. The researcher found that 80% of the patients had poor medication adherence, and 21.3% prevalence were younger patients with comorbidities compared with patients who complied with their prescribed medication. The researcher discovered that clients with poor medication adherence had higher level of lipoprotein cholesterol, and the rate of re‐hospitalization among these types of patients was very high. Therefore, they established that there is a significant relationship between medication adherence and the associated complications (American Diabetes Association, 2008). Therefore, there is a need to find the factors responsible for poor medication adherence among patients with diabetes. Poor medication adherence could have serious health burden such as increased mortality rate, decrease in glycemic control, hospitalization and other complications (American Diabetes Association, 2010).

Sweileh et al. (2014) found out in their study that the medication beliefs of client concerning the importance of taking prescribed medications for the treatment of their health condition and its consequences will definitely affect their attitude towards medication adherence. They postulated that clients with diabetes who had positive belief towards medication and the importance of antidiabetic medications were more likely to comply with the prescribed medications. However, clients with diabetes who believed that diabetic medications had adverse effect on their system and saw that regimen has been harmful were more prone to poor medication adherence (Sweileh et al., 2014). Thus, it could be deduced that there is a correlation between medication adherence and medication belief.

### 1.1 Statement of problems

The World Health Organisation (2015) projected that over 366 million people are affected with diabetes mellitus worldwide and many are ignorant of having the disease. According to World Health Organisation (2015), diabetic cases had tripled in the last two decades with the highest prevalence rates in developing countries such as Nigeria. In Nigeria, apart from HIV‐AIDS, Ebola virus and lassa‐fever which are infectious disease, diabetes is the major disease of Nigerians (Bos & Agyemang, 2013). The government and other non‐governmental agencies such as the Diabetic Association of Nigeria (DAN) put several programmes in place to reduce the incidence of the disease, but minimal progress has been recorded. This could be attributed to the reasons why majority of discharged patients with diabetes often return to the hospital within a short time, either for same diabetes crisis, comorbidities or for related complications. An empirical finding showed that there are increased rates of re‐admission among patients with diabetes associated with poor medication adherence after discharge and emphasized on the need to involve the clients on personal discussion on their health condition and why they need to improve their level of participation in their care (Sharma, Kalra, Dhasmana, & Basera, 2014). There are possibilities that these patients do not have enough knowledge about the complications that could arise from poor management or refusal in taking treatment as prescribed. It could also be that their cultural beliefs and current economic situations are causing poor medication adherence. Therefore, the researcher is interested in correlating medication belief, with medication adherence among patients with diabetes mellitus.

## 2 THE STUDY

### 2.1 Study setting

The study was set in Edo State, Nigeria. The research population is made of all patients with diabetes who are registered with three hospitals in Benin‐city, in the following orders: University of Benin Teaching Hospital (*N* = 90), Central Hospital Benin (*N* = 50) and Faith Mediplex (*N* = 40). The healthcare system is financed by the Federal government, State government and Missionary. These three aspects were included in this study.

### 2.2 Study design

A correlational research design was adopted, using standardized structured questionnaire to elicit information from the respondents. This enabled the researcher in determining association between the medication belief and medication adherence among patients with diabetes mellitus.

### 2.3 Participants

A total enumeration was adopted for the study, where all the registered adult patients were used for the study. Total enumeration was more appropriate because the size of the population is not too large and was within a manageable size for the study.

### 2.4 Instrumentation

#### 2.4.1 Beliefs about medicines questionnaires (BMQ)

A belief about medicines questionnaires is a standardized scale that is often used by researchers to measure the beliefs of patients about drug and other medication care. In this study, the belief of clients with diabetes about diabetic medications was assessed through the test. The instrument has 18 items that assessed the health beliefs of patient and is broadly divided into specific and general. The specific part is used specifically, to determine the beliefs of clients with diabetes about prescribed diabetic medications and is made up of two scales which are used to determine belief about necessity and concern of the client about the prescribed diabetic medications. This part of the scale is made up of five items, while the general part is used to determine the general beliefs of client with diabetes about medication and is also made up of two scales, which are as follows: the General over use and General‐harm scale, which assesses the general perception of clients about how medications are combined for their treatment and perceived harm, respectively. The research instrument was structured in five‐point scale which was scored as follows: strongly disagree, disagree, uncertain, agree and strongly agree and will have a score of 5 to 1, respectively.

This test is rated as follows: at the specific part, the BMQ score ranges from 5‐25 score. At this point, if the necessity scores are higher than concern scores, it means that the client belief that adherence to the medication will promote quick recovery, but when concern score is greater than necessity, the clients have a negative belief about the prescribed medication and the tendency of the client not to adhere with the medication is very high. Moreover, the general part of the scale, the BMQ scores ranges from 4 to 20. In this area, if the general‐overuse score is higher than the general‐harm scales, it means that the client has a negative view about how the physician combined the medication for their treatment and believe that they are overused, but when general‐harm score is greater than general‐overuse, it means that the clients have negative perception about medication and believe that medication has the tendency of causing harm or poison their system.

### 2.5 Data collection procedure

The researcher established the rule of inclusion and exclusion to identify the qualified participants for the study, and all available registered patients with diabetes in the diabetic clinic who meet up with the rules of inclusion were selected from the three hospitals in Benin‐city. The selected clients were invited to join the study voluntarily and informed verbal consents were obtained, after explaining the importance of the study. The participants were asked to complete the research instrument on: (a) Morisky Medication Adherence test to find out the level of medication adherence among clients with diabetes; and (b) Beliefs about Medicines test; this is to determine the beliefs of clients with diabetes about diabetic medications.

### 2.6 Data analysis

To analyse the data, descriptive statistics such as frequency and percentage and inferential statistics such as Cronbach correlation coefficient were used.

## 3 FINDINGS

Table 1 shows that out of 180 respondents: concerning age, eight (4.4%) were in age group of 27–36 years, 49 (27.2%) were in age group of 37–46 years, 30 (16.7%) were in age group of 47–56 years, 39 (21.7%) were in age group of 57–66 years, 43 (23.9%) were in age group of 67–76 years and 11 (6.1%) were in age group of 77 years and above. Mean age was 57.18, median = 60, mode = 75 and standard deviation (*SD*) 14.15. Regarding sex of the respondents, 80 (44.4%) were male and 100 (55.6%) were female. Concerning the marital status of the respondents, 90 (50%) were married, 70 (38.9%) were single and 20 (11.1%) were divorced. Regarding a stream of the education level of the respondents, 10 (5.6%) had no formal education, 64 (35.6%) had primary education, 77 (42.8%) had secondary education and 29 (16.1%) had a college education. On economic and income level, the table shows that 92 (51.1%) monthly salary is less than 75,000 naira, 66 (36.7%) earned between 75,000 and 100,000 and 22 (12.2%) earned above 100,000 naira monthly. Table 2 shows the level of medication adherence among 180 respondents with diabetes. One hundred (55.6%) of the participants had low adherence level, 70 (38.9%) of the participants had medium adherence level and 10 (5.6%) of the participants had higher adherence level. This analysis showed that majority of the participants had a low level of medication adherence.

**Table 1:** Demographic characteristic of the despondence

Variables	Frequency	Percent	Valid percent	Cumulative percent
Age (years)				
27–36	8	4.4	4.4	4.4
37–46	49	27.2	27.2	31.6
47–56	30	16.7	16.7	48.3
57–66	39	21.7	21.7	70.0
67–76	43	23.9	23.9	93.9
77 & Above	11	6.1	6.1	100.0
Mean = 57.18, median = 60, mode = 75 and standard deviation = 14.15				
Sex				
Male	80	44.4	44.4	44.4
Female	100	55.6	55.6	100.0
Marital status				
Married	90	50.0	50.0	50.0
Single	70	38.9	38.9	88.9
Divorce	20	11.1	11.1	100.0
Education				
No formal education	10	5.6	5.6	5.6
Primary	64	35.6	35.6	41.1
Secondary	77	42.8	42.8	83.9
College	29	16.1	16.1	100.0
Economic and income level				
Less than 75,000	92	51.1	51.1	51.1
75,000–100,000	66	36.7	36.7	87.8
Greater than 100,000	22	12.2	12.2	100.0

**Table 2:** Shows the frequency distribution of medication adherence level among patients with diabetes

	Medication adherence			
	Frequency	Percent	Valid percent	Cumulative percent
Low adherence	100	55.6	55.6	55.6
Medium adherence	70	38.9	38.9	94.4
High adherence	10	5.6	5.6	100.0
Total	180	100.0	100.0	

Table 3 rated the scores as follows: at the specific part, the BMQ score ranges from 5‐25. At this point, the concern mean score is greater than necessity means scores, and this means that the clients have a negative belief about the prescribed medication and the tendency of the client not to adhere to the medication could be very high. Moreover, at the general part of the scale, the BMQ scores range from 4‐20. At this area, the general‐harm mean score is greater than general‐overused mean scores, and this could mean that the clients have a negative perception about medication and believe that medication has the tendency of causing harm or poison their system, meaning that the tendency of the client presenting with low medication adherence will be very high. Table 4 shows that the Spearman's rho value of −0.208 and a *p*‐value of 0.005. Testing at an alpha level of 0.05, the *p*‐value is lesser than the alpha level, so the null hypothesis which states that there is no significant correlation between medication belief and medication adherence is rejected. Consequently, there is a significant correlation between medication belief and medication adherence. So, this means that if patients with diabetes have positive belief about their drugs, their medication adherence level will also increase.

**Table 3:** Descriptive statistics showing the medication belief of patients with diabetes

	*N*	Minimum	Maximum	Mean	Std. Deviation	Variance	Skewness	Std. Error
	Statistic	Statistic	Statistic	Statistic	Statistic	Statistic	Statistic	
S								
Necessity	180	19	24	20.71	1.034	1.069	1.352	0.181
Concern	180	19	24	22.23	1.182	1.398	−0.196	0.181
G								
General‐overuse	180	13	20	16.01	1.615	1.069	0.474	0.181
General‐harm	180	14	20	16.95	1.702	1.398	−0.251	0.181
Total	180							

Note

*N*: total number of participants; S: specific part and G: general part of the Beliefs about Medicines Questionnaires (BMQ)

**Table 4:** Correlation between medication belief and medication adherence among patient with diabetes

Variables	*N*	Spearman's rho	Sig. (2‐tailed)	Remarks
Medication adherence				
Medication belief	180	−0.208	0.005	Sig.

Note

*α* *=* *0.05*

## 4 DISCUSSION

The result of the present study indicated that majority of the participants had a low level of medication adherence. These findings agreed with Ahmad, Ramli, and Paraidathathu (2013) who stated that the medications adherence level in patients with diabetes is greatly below average and this is associated with lots of life‐threatening complications that increase the cost of management with optimal health outcomes and identified the factors that can predispose patients with diabetes to medication non‐adherence as the patient's age and level of health literacy. Cramer (2014) in his own study also supported this view because he discovered that between 36% and 93% of patients with diabetes did not keep to the prescription diabetic medications therapy for several months.

The result of the present study showed that there is a relationship between patient belief and medication adherence. It also shows that patients with diabetes have a negative belief about the prescribed medication, negative perception about medication and believe that medication has the tendency of causing harm or poisoning their system. The study revealed that most of the people in Benin‐city believe in traditional medicine and most times, you find them saying that orthodox medicine can only cure the disease of the whites, while herbs cure diseases of the black and you hardly find them coming to the hospital until the case is bad. The result of the present study is consistent with the study of DiMatteo (2011), who carried out an extensive search of published articles from 1948‐2005 which resulted into 116 articles that are relevant to medication adherence and found out that there is a significant correlation between patient belief about diseases and treatment and medication adherence. This is further supported by a research carried out by Horne, Chapman, and Parham (2013), which found that patients’ beliefs about treatment influence their concern and judgements about the benefit or potentially adverse effect that can be associated with intake of prescribed medication.

### 4.1 Study limitations

This study was conducted at a single state that had culture that does not support modern medicine; such may have influenced the result. At the same time, the sample size was small as recommended in grounded theory since this is a part of a larger study that includes quantitative measures. Furthermore, the cost and availability of drugs are also major factors that may have influenced the study.

## 5 CONCLUSION

The study concluded that medication adherence among patients with diabetes is very low characterized with negative belief towards their drugs and also concluded that there is a relationship between patient belief and their medication adherence level. Therefore, to improve on medication adherence level, patients’ belief must be worked on.

### 5.1 Recommendations

Based on the discoveries of the study, the recommendations were prepared as follows: public education campaigns regarding compliance with medication should be encouraged and nursing care for patient with diabetes need to pay more attention on health belief of their client. I therefore suggest that nurses should empower patients to always ask questions, and this is important to correct negative medication beliefs; lastly, I recommend additional research on medication adherence and medication belief, to explore reasons why patients with diabetes have such beliefs about their drugs.

## ETHICAL STATEMENT

The study was passed through the research and ethical committee of University of Benin teaching hospital, faith medication complex and Ministry of Health in Edo state for approval. The interviewer explained the importance and what the participants and others stand to benefit from the study. Therefore, obtained informed consent from the participants before the study commences. The participation in the study was voluntary, and they have right to pull out from the study at any level as the project progresses, is the choice of the participants, and without any harmful effect on the subject. The researcher ensured strict confidentiality by assuring anonymity in coding and interpretation of data throughout the study. The study obeyed all the rules and regulation of ethical guidelines in research and was also approved by Babcock University ethical and research committee.

## CONFLICT OF INTEREST

There is no conflict of interest declared by the author.
